# Synergistic Anticandidal Effects of Six Essential Oils in Combination with Fluconazole or Amphotericin B against Four Clinically Isolated *Candida* Strains

**DOI:** 10.3390/antibiotics10091049

**Published:** 2021-08-27

**Authors:** Bouchra Soulaimani, Elena Varoni, Marcello Iriti, Nour-Eddine Mezrioui, Lahcen Hassani, Abdelaziz Abbad

**Affiliations:** 1 Laboratory of Microbiology and Biotechnology, Agrosciences and Environment, Faculty of Sciences Semlalia, Cadi Ayyad University, Marrakech 40000, Morocco; bouchrasoulaimanigebc@gmail.com (B.S.); mezrioui@uca.ac.ma (N.-E.M.); lhassani@uca.ac.ma (L.H.); abbad.abdelaziz@gmail.com (A.A.); 2 Department of Biomedical, Surgical and Dental Sciences, Università degli Studi di Milano, 20142 Milan, Italy; elena.varoni@unimi.it; 3 Department of Agricultural and Environmental Sciences, Università degli Studi di Milano, 20133 Milan, Italy

**Keywords:** essential oils, anticandidal activity, *Candida* spp., synergy

## Abstract

The development of opportunistic pathogenic *Candida* strains insensitive to several classes of antifungals has emerged as a major health care problem during the last years. Combinational therapy of natural products (e.g., essential oils, EOs) with conventional antifungals has been suggested as a promising alternative to overcome this medical problem. The present study investigates the potential antifungal activity of EOs extracted from some selected medicinal plants, alone and in combination with two common conventional antifungals (fluconazole and amphotericin B) against four clinical *Candida* isolates. MIC assays indicated that EOs induced strong anticandidal activities with MIC values ranging from 0.162 to 4.950 mg/mL. The combination of amphotericin B with *Thymus leptobotrys*, *Origanum compactum* and *Artemisia herba alba* EOs provided a synergistic effect against *C. krusei* only, with MIC gain of four-fold, and additive effect against remaining strains (MIC gain = two-fold). Interesting synergistic interactions were observed by combining all studied EOs with fluconazole, with reduction rates of their MICs ranging from 16 to 512-fold. This synergistic effect was very pronounced with the combination of *T. leptobotrys* EO and fluconazole. These findings indicate that studied EOs can be used as anti-candidals in combination with antifungals, particularly fluconazole, to counteract the emergence of resistant *Candida* spp.

## 1. Introduction

The incidence of systemic fungal infections has been increasing significantly and currently affects millions of people worldwide [1,2,3]. Yeasts of Candida genus are reported to be responsible for 80% of fungal infections, which are recognized as one of the most common nosocomial contaminations, producing important morbidity and mortality rates, particularly in immunocompromised patients [4,5]. Within 200 yeast species, *Candida albicans* has been described as the most common pathogen found in severe candidiasis infections, but other non-albicans *Candida* spp. such as *C. glabrata, C. tropicalis, C. krusei,* and *C. parapsilosis* are becoming increasingly insidious [6,7,8]. The current treatment of these candidiasis infections remains essentially based on the use of common polyenes (e.g., amphotericin B) and azole antifungals (e.g., fluconazole), mainly targeting ergosterol in the fungal cell membrane or its biosynthetic pathway [9,10,11]. However, the fungistatic property of many of these antifungals, in addition to the increased therapy cost and the emergence of clinical drug resistance, limit their success in clinical practice [9,10,12,13]. The development of novel antifungal agents is becoming difficult and challenging due to the eukaryotic nature of *Candida* cells, which results in a limited number of drug targets. The required antifungals should be specific against the pathogen’s targets, which are not shared with human hosts; otherwise, these antifungals should display a selective toxicity to the fungal cell, while being safe towards the human host cell [14]. Alternatively, the synergy between conventional drugs and natural antimicrobial products has been described as an emerging strategy to minimize the effective doses of standard antifungals, minimizing their side effects and their related toxicity, while enhancing their biological efficacies [15,16]. Among natural products, essential oils (EOs) from medicinal plants constitute rich sources of bioactive compounds with strong antimicrobial activities and low cytotoxicity against the host [17]. Several EOs have been reported to present high synergistic interactions with conventional antimicrobials against several pathogenic micro-organisms, including polyene and azole-resistant *Candida* isolates [15,18,19]. In fact, many EO components target multiple metabolic pathways in *Candida* cells, which can overcome or delay the emergence of drug resistance [19,20,21]. Additionally, some terpenoids, when used in combination, are able to transform the fungistatic nature of fluconazole into a fungicidal drug, and to inhibit the antifungal efflux by blocking drug transporter pumps [15,18].

*Thymus leptobotrys* Murb. (Lamiaceae), *T. pallidus* Batt. (Lamiaceae), *T. satureioides* Coss. (Lamiaceae), *Origanum compactum* Benth. (Lamiaceae), *Artemisia herba alba* Asso. (Asteraceae) and *Ammodaucus leucotrichus* (Coss. &Durieu) (Apiaceae) are extensively used in Moroccan folk medicine in different forms to treat many fungal diseases [22]. Moreover, their antibacterial, antifungal, antiparasitic and antiviral activities have been reported in many previous studies [23,24,25,26,27,28]. To the best of our knowledge, no study has yet been published about synergistic interactions of their EOs with conventional antifungals. Therefore, the aim of the present work was to evaluate the capacity of these EOs to enhance the anticandidal effects of amphotericin B and fluconazole, when used in combination at significantly low concentrations, against four clinically isolated fungal strains, namely *C. albicans*, *C. glabrata*, *C. krusei* and *C. parapsilosis*.

## 2. Results and Discussion

### 2.1. Chemical Composition of the EOs

The EOs extracted by steam-distillation were found to be pale to dark yellow, except for *A. leucotrichus* EO which was blue, with yields ranging from (0.63 ± 0.05)% to (2.15 ± 0.02)% (*v*/*w*) based on dry weight (Table 1). Generally, the EO yields of dried aerial parts of *T. pallidus* and *T. leptobotrys* were similar to those reported previously [28,29], while the fruit of *A. leucotrichus* and the aerial parts of *T. satureioides*, *O. compactum* and *A. herba alba* yielded a lower number of EOs compared to those obtained in many previous works [23,25,26,27,29]. The results of the chemical analysis of the volatile constituents of the EOs (percentage content of each compound, elution order, retention index (RI), Retention time (RT) and structural subclass) are summarized in Table 2. Sixty-eight constituents were identified, which accounted for 94.34–99.20% of the total oils. Generally, the studied EOs were quantitatively dominated by oxygenated monoterpenes (41.65–92.64%), except *T. pallidus* EO which was dominated by monoterpene hydrocarbons (57.70%). GC-MS analysis revealed a high content of carvacrol (78.75%) in *T. leptobotrys* EO, which is in agreement with that previously reported in the literature [29,30]. The main constituents of *T. pallidus* EO were found to be γ-terpinene (29.6%), thymol (26.8%) and p-cymene (18.9%) (Table 2), which is in accordance with those reported in many previous works [31,32]. However, many other chemotypes dominated by camphene (7.5–17.7%), myrcene (1.1–15.4%) and camphor (28.5–29.8%) have been reported in *T. pallidus* EOs originating from other Moroccan regions [30,33]. The EO of *O. compactum* was dominated by carvacrol (35.69%), p-cymene (13.72%) and carvacrol methyl ether (11.69%). This chemical profile is similar to that obtained for EOs of some *O. compactum* samples harvested from different regions of northern Morocco [34]. Otherwise, γ-terpinene (8.72–17.25%) thymol (10.33–15.75%) and p-cymene (8.44–18.59%) were identified, beside carvacrol (43.58–47.85%), as main oil constituents of the plant in other Moroccan regions [25,35]. The main oil constituents from *T. satureioides* were carvacrol (25.45%), borneol (13.66%) and caryophyllene (12.10%), which is similar to what has been previously reported in the literature [31,33,36,37]. However, camphene (11.8%) and α-terpineol (10.4%), beside borneol (26.7%), were reported as major constituents of *T. satureioides* EO originating from the Imouzzer region [30]. Concerning *A. leucotrichus* EO, the main compounds were found to be l-perillaldehyde (46.63%), D-limonene (23.81%) and bornyl angelate (6.24%). This chemical profile is similar to those reported in previous studies, where perillaldehyde and limonene were found to be the major components of *A. leucotrichus* EO, while bornyl angelate was absent or present at a low concentration [38,39,40].

*A. herba alba* EO was dominated by cis-thujone (42.40%), trans-thujone (28.77%) and camphor (16.65%). This composition is qualitatively similar to that reported previously in some studies, but with some quantitative differences [41,42,43]. In contrast, another chemical profile dominated by chrysanthenone (47%) camphor (24%) and verbenone (7.2%) was reported for the EO of *A. herba alba* collected from Tahanaoute region [27].

### 2.2. Anticandidal Activity

The anticandidal activities of studied EOs towards Candida strains were evaluated using broth microdilution assays. The results given in Table 3 demonstrated that all EOs expressed strong anticandidal effects with MIC values ranging from 0.162 mg/mL to 4.950 mg/mL (Table 3). *A. leucotrichus*, *O. compactum* and *T. leptobotrys* EOs displayed the strongest inhibitory activity against all tested Candida strains, with comparative MIC values ranging from 0.162 mg/mL to 0.596 mg/mL. These values were, interestingly, lower than those of fluconazole (MIC = 1 mg/mL for all Candida strains). EOs of *T. saturioiedes* and *T. pallidus* expressed high anticandidal activities at concentrations ranging from 0.373 mg/mL to 2.598 mg/mL, while *A. herba alba* EO showed poor effect towards all tested strains, with MIC values between 2.475 mg/mL and 4.950 mg/mL.

The relatively strong activity of *A. leucotrichus* observed in our study is in line with those works reporting the effect of this oil on some Candida strains [28,44,45,46]. This strong activity can be mainly explained by the presence of high content of perillaldehyde, an oxygenated monoterpene previously tested for its potent antimicrobial activity [47,48,49]. *O. compactum* and *Thymus* species have long been known for their large spectrum of activity against numerous pathogenic strains [23,25,50]. Their antifungal effect is attributed to the presence of bioactive antimicrobial compounds, especially carvacrol, thymol, borneol, p-cymene and γ-terpinene. The anticandidal effect of these monoterpenoids has been well demonstrated, with carvacrol being more active than the other thyme oil constituents [15,51,52,53], and can explain the relative high activities of *T. leptobotrys* and *O. compactum* which contain higher percentages of this phenolic monoterpene compared to the remaining studied thymes. The moderate anticandidal effect of *A. herba alba* obtained in our study is consistent with that previously reported [41].

### 2.3. Synergistic Effect of EOs with Conventional Antifungals

The results of the synergistic interactions (FICI values and MIC gain of the antifungals) between studied EOs and conventional antifungals fluconazole and amphotericin B are reported in Table 4 andTable 5, respectively. The combinations of studied EOs and fluconazole gave very pronounced synergistic effects regarding all tested Candida strains, with FICI values between 0.25 and 0.31 (Table 4). Among these combinations, the one prepared by *T. leptobotrys* EO showed the greater synergism, with promising reduction in fluconazole MICs of *Candida* strains up to 512 fold. The addition of *T. satureioides* or *T. pallidus* EOs reduced the fluconazole MICs for *C. albicans*, *C. glabrata* and *C. krusei* by 456 fold and by 64 fold for *C. parapsilosis*. EOs of *O. compactum* and *A. leucotrichus* decreased the MIC values of fluconazole 128 fold for *C. albicans* and *C. glabrata*, 64 fold for *C. krusei* and 16 fold for *C. parapsilosis*, while *A. herba alba* EO reduced the fluconazole MICs for *C. albicans*, *C. glabrata* and *C. krusei* by 64 fold and *C. parapsilosis* by 32 fold. From these results, it can be observed that the synergistic effect of EOs with fluconazole was stronger than that obtained with amphotericin B (Table 5). Indeed, the combination of amphotericin B with sub-MICs of *T. leptobotrys*, *A. herba alba* or *O. compactum* EOs gave synergistic effects against *C. krusei* only, with FICI = 0.5 and four-fold gains, while additive effects (FICI = 0.75) and gains of two-fold were obtained against the other tested Candida strains. Remaining EOs gave MIC gains of 1 towards all tested Candida and FICI = 1.25, indicating indifferent outcome. Interestingly, for all combinations and for the two conventional antifungals studied, none was found to be antagonistic against the tested strains.

To the best of our knowledge, the combinations of the EOs and antifungals (fluconazole and amphotericin B) have not been investigated previously. However, the studied EOs are characterized by the presence of some major compounds known to possess strong antimicrobial effects and synergism interactions with many antimicrobials [20,54,55]. In fact, carvacrol and thymol (principal components of studied *Origanum* and *Thymus* EOs) were reported to interact with the cytoplasmic membrane by its entry between the acyl chains of phospholipids, leading to the disruption of its fluidity and permeability [20,56,57]. This mechanism may facilitate the permeability of fluconazole through the fungal membrane to the intracellular target, acting together on the ergosterol biosynthesis pathway. Carvacrol and thymol were reported to increase the effectiveness of fluconazole by chemo-sensitizing the Candida cells to the antifungal and decreasing its extrusion by efflux pumps [15].

## 3. Materials and Methods

### 3.1. Plant Material and EOs Extraction

Aerial parts of T. *leptobotrys, T. pallidus, T. satureioides, O. compactum, A. herba alba* and fruits of *A. leucotrichus* were collected from different wild locations in Morocco (Table 1) and identified by one of the authors (Abbad. A). Plants collected were dried in the shade at room temperature (≈25 °C) and voucher specimens were deposited at the Laboratory of Microbial Biotechnologies, Agrosciences and Environment of the Faculty of Science Semlalia, University Cadi Ayyad, Marrakech. Each dried plant material was submitted to four successive steam-distillations (4 × 200 g) for about 3 h using a Clevenger-type apparatus, and recovered EOs were dried with anhydrous sodium sulfate and stored in hermetically sealed vials at 4 °C until use.

### 3.2. GC/MS Analysis

Qualitative and quantitative analysis of the EO chemical compounds was carried out using a gas chromatograph equipped with a TG-5MS column (length: 30 m; internal diameter: 0.25 mm, thickness film: 0.25 mm) and coupled to a mass selective detector ISQ (Single Quadrupole Mass spectrometer). The carrier gas was helium with flow rate of 1.0 mL/min. EO samples (20 µL) were diluted in 2 mL of hexane and 2.0 μL of the dilution was injected, using split mode. The injection temperature was 260 °C and the column temperature was programmed from 100 to 260 °C at a rate of 4 °C/min with a hold of 10 min at 246 °C. The transfer line and ion source temperatures were held at 230 °C with EI ionization (70 eV) and the m/z scan range was of 41–500. Identification of the individual components was carried out by matching their mass spectra with WILEY275, NBS75K, and Adams terpene library [58], and standards of the main components where possible. For semi-quantification purposes, the normalized peak area of each compound was used without any correction factors to establish abundances.

### 3.3. Candida Strains

The yeast strains used in this study were provided by the Moroccan coordinated collection of microorganisms. *C. albicans* (CCMM L4), *C. glabrata* (CCMM L7), *C. krusei* (CCMM L10) and *C. parapsilosis* (CCMM L18) are clinically isolated fungal strains originating from patients suffering from acute candidiasis. They were cultured in Sabouraud Dextrose Agar at 28 °C for 48 h [59].

### 3.4. Determination of the Minimum Inhibitory Concentration (MIC)

The anticandidal activities of EOs were evaluated using broth microdilution method according to the Clinical and Laboratory Standards Institute (CLSI) guidelines M27-A3 [60]. EO dilutions were performed in Sabouraud Dextrose Broth supplemented with dimethyl-sulfoxide (DMSO), at a final concentration of 1%, to enhance oil solubility. A negative control was prepared using the same concentration of DMSO. Then, 100 μL of each dilution was mixed with the same volume of cell suspension at 1–2 × 10^3^ cells/mL. The microplates were incubated at 28 °C for 48 h and the MIC value was defined as the lowest EO concentration that inhibits macroscopic growth of the tested strains. Fluconazole and amphotericin B were used as positive controls.

### 3.5. Synergistic Effect of EOs with Conventional Antifungals

Synergistic interactions between conventional antifungals (fluconazole and amphotericin B) and EOs, were determined using microdilution assay [16]. Briefly, 50 μL of a serial dilution of antifungals (from MIC to 1/512 MIC) were added to microwells containing 50 μL of the EO at sub-inhibitory concentrations (1/4 MIC), and previously seeded by 100 μL of cell suspensions. The microplates were incubated at 28 °C for 48 h. The MIC values of antifungals in combination with sub-MICs of EOs were determined and fractional inhibitory concentration index (FICI) was calculated using the following formula: FICI = FIC of EO + FIC of antifungal

FIC of EO = MIC of EO in combination with antifungal/MIC of EO alone, and FIC of antifungal = MIC of antifungal in combination with EO/MIC of antifungal alone.

The FICI results were interpreted as: A synergism when (FICI ≤ 0.5), additive effects when (0.5 < FICI ≤ 1), indifference when (1 < FICI ≤ 2) or an antagonism when (FICI ≥ 2) [61].

The MIC gain of the antifungal was determined as MIC of antifungal alone/MIC of antifungal in combination with EO.

## 4. Conclusions

The present work provides new information regarding the anticandidal potential of some selected Moroccan EOs and their synergistic effects with two common antifungals (fluconazole and amphotericin B). The results indicate that all EOs studied possess anticandidal activity, with those extracted from *A. leucotrichus*, *O. compactum* and *T. leptobotrys* being more effective. The addition of the different EOs at sub-inhibitory concentration reduces the fluconazole and amphotericin B MICs of the tested *Candida* strains by 16 to 512-fold and one to four-fold, respectively. Among the EOs examined, *T. leptobotrys* EO combined high anticandidal effect and high synergistic interaction with two conventional antifungals, mainly fluconazole. These findings showed that the studied EOs, in particular *T. leptobotrys* EO, may be used as effective anticandidal agents to restore the efficacy of these common antifungal drugs for combating resistant-*Candida* strains. Additional studies are necessary to determine the mechanism of these synergistic antifungal associations.

## Figures and Tables

**Table 1:** Local names, harvesting location and period, voucher specimens and EO yield for the studied plants.

Plant Species	Local Names	Harvesting Place	Harvesting Time	Voucher Specimens	Latitude/Longitude	Oil Yields ^a^ (%)
*T. leptobotrys*	Za-itra	Tafraoute	May 2019	TL-03	29°42′ N/08°74′ W	1.85 ± 0.07
*T. saturioiedes*	Za-itra	Idni	May 2019	TS-06	30°54′ N/08°17′ W	0.66 ± 0.08
*T. pallidus*	Za-itra	Ait Lkak	June 2019	TP-13	31°17′ N/07°50′ W	2.15 ± 0.02
*O. compactum*	Zaatar	Toufliht	June 2018	OC-12	31°28′ N/07°32′ W	0.63 ± 0.05
*A. leucotrichus*	Kemoune essoufi	Tata	June 2018	AL-17	29°44′ N/07°54′ W	1.25 ± 0.07
*A. herba alba*	Sheeh	Ijoukak	September 2019	AHA-18	30°59′ N/8°09′ W	0.81 ± 0.01

^a^ Yield of EOs determined based on their volume/weight of the sample used for distillation.

**Table 2:** Chemical compounds of studied EOs.

RT ^a^	RI ^b^	Compounds ^c^	Tl	Tp	Ts	Oc	Al	Aha
2.70	928	α-Thujene	0.29	1.40	0.31	0.41	- ^d^	-
2.78	931	α-Pinene	0.26	1.30	2.82	0.52	4.20	-
2.92	950	Camphene	-	2.20	4.11	0.14	0.15	1.63
3.02	975	1-Octen-3-ol	0.10	0.30	-	0.22	-	-
3.10	983	3-Octanone	0.11	-	-	-	-	-
3.12	989	Sabinene	-	-	-	-	0.16	-
3.17	992	Myrcene	0.71	0.90	1.89	1.21	-	-
3.41	1005	α-Phellandrene	-	-	-	0.13	-	
3.52	1019	α-Terpinene	-	2.70	0.75	1.26	-	-
3.62	1027	*p*-Cymene	1.91	18.90	9.50	13.72	-	0.64
3.67	1030	d-Limonene	-	0.70	-	-	23.81	-
3.73	1031	1.8-Cineole	-	-	-	-	-	0.85
3.81	1047	Ocimene	-	-	-	0.30	-	
4.03	1057	γ-Terpinene	1.44	29.60	6.70	8.97	0.91	-
4.17	1067	*trans*-Sabinene hydrate	0.40	-	-	0.51	-	-
4.53	1098	Linalool	0.52	4.70	7.79	2.37	0.41	-
4.78	1108	*cis*-Thujone	2.56	-	-	-	-	42.40
4.95	1118	*trans*-Thujone	1.98	-	-	-	-	28.77
5.09	1127	Chrysanthenone	-	-	-	-	-	0.91
5.50	1146	Camphor	0.62	-	-	-	0.42	16.65
5.87	1169	Borneol	0.64	5.40	13.66	0.40	0.25	1.72
6.06	1180	l-terpinen-4-ol	0.55	-	0.98	0.46	-	0.65
6.14	1188	α-Terpineol	-	-	3.95	1.02	-	
6.29	1197	Caranone	-	-	0.33	-	-	-
6.42	1200	Dihydro-carvone	0.18	-	0.20	-	-	-
6.74	1213	Verbenone	-	-	-	-	-	0.69
7.14	1230	Thymol methyl ether	-	-	-	0.12	-	-
7.36	1241	Cumin-aldehyde	-	-	-	-	3.83	-
7.37	1244	Carvacrol methyl ether	-	-	-	11.69	-	-
7.54	1257	Linalyl acetate	-	-	-	-	0.84	-
7.78	1260	Chrysanthenyl acetate	0.12	-	-	-	-	1.00
8.22	1275	l-Perillaldehyde	-	-	-	-	46.63	-
8.39	1286	1.4-p-Menthadien-7-al	-	-	-	-	1.73	-
8.40	1288	Thymol	0.85	26.8	-	1.20	-	-
8.41	1289	Bornyl acetate	-	-	1.32	-	-	-
8.54	1290	2-Caren-10-al	-	-	-	-	1.53	-
8.68	1301	Perrilla alcohol	-	-	-	-	0.28	-
8.69	1303	Carvacrol	78.75	1.40	25.45	35.69	-	0.30
9.05	1329	2-Methoxy-4-vinylphenol	0.32	-	-	-	-	-
10.50	1366	Carvacrol acetate	0.21	-	-		-	-
10.67	1370	Ylangene	-	-	-	0.13	-	-
10.78	1375	α-Copaene	-	-	-	0.55	-	-
11.05	1389	(−)-β-Bourbonene	-	-	-	0.26	-	-
11.18	1397	Methyl perillate	-	-	-	-	1.51	-
11.68	1413	α-Gurjenene	-	-	0.10	-	-	-
11.98	1415	Caryophyllene	1.60	2.90	12.10	4.09	0.30	-
12.21	1425	β-Gurjunene	-	-		0.24	-	-
12.35	1445	Aromandendrene	0.62	-	0.11	1.60	-	-
12.50	1452	Humulene	-	-	0.64	0.42	-	-
13.07	1462	epi-β-Caryophyllene	0.15	-	-	0.12	-	-
13.09	1464	γ-Decalactone	-	-	-	-	0.19	-
13.45	1484	γ-Murolene	-	-	-	1.02	-	-
13.62	1489	Germacrene D	0.26	-	-	0.42	0.27	-
14.00	1500	Viridi-florene	0.56	-	-	1.63	-	-
14.16	1504	β-Himachalene	-	-	-		0.57	-
14.25	1511	β-Bisabolene	-	-	-	1.53	-	-
14.48	1518	Cubebol	0.11	-	-	-	-	-
14.49	1519	γ-Cadinene	-	-	0.51	0.82	-	-
14.71	1526	Cadina-1(10).4-diene	0.21	-	-	2.16	-	-
15.11	1531	α-Cadinene	-	-	-	0.17	-	-
15.74	1550	Bornyl angelate	-	-	-	-	6.24	-
16.23	1580	(+)-Spatulenol	0.33	-	-	0.86	-	-
16.34	1589	*cis*-Davanone	0.73	-	-	-	-	2.34
16.39	1591	Viridiflorol	0.39	-	-	-	-	-
16.63	1598	Caryophyllene oxide	-	-	1.13	1.03	-	-
17.89	1649	*τ*-Cadinol	-	-	-	-	0.64	-
18.97	1685	α-Bisabolol	-	-	-	-	0.33	-
19.26	1700	Shyobunol	-	-	-	-	0.83	
Oxygen-containing monoterpenes			87.05	38.30	52.03	41.65	55.27	92.64
Monoterpene hydrocarbons			4.61	57.70	26.07	26.66	29.23	2.27
Oxygen-containing sesquiterpenes			1.56	0.00	1.13	1.89	8.04	2.34
Sesquiterpene hydrocarbons			3.40	2.90	13.46	15.16	1.14	0.00
Other			0.86	0.30	1.58	12.03	2.35	1.00
Total			97.48	99.20	94.34	97.39	96.03	98.25

Tl: *T. leptobotrys,* Tp: *T. pallidus,* Ts: *T. satureioides,* Oc: *O. compactum,* Al: *A. leucotrichus,* Aha: *A. herba alba*. ^a^ RT retention times. ^b^ Retention indices determined using the homologous series of n-alkanes. ^c^ Compounds listed in order of elution.^d^ Not detected.

**Table 3:** Minimal inhibitory concentrations (MIC) of essential oils and two conventional antifungals (mg/mL).

Microorganisms	Tl ^a^	Ts	Tp	Oc	Al	Aha	Fluconazole	Amphoterecin B
*C. albicans*	0.596	0.598	2.598	0.278	0.324	2.475	1	0.0001
*C. glabrata*	0.297	0.373	0.644	0.278	0.162	4.950	1	0.0001
*C. krusei*	0.297	1.196	1.299	0.278	0.162	4.950	1	0.0001
*C. parapsilosis*	0.297	1.196	0.644	0.278	0.162	4.950	1	0.0004

^a^ The abbreviations of the species are given in Table 2.

**Table 4:** Fractional inhibitory concentrations indices (FICIs) and gain of fluconazole combined with the essential oils.

EOs	*C. albicans*			*C. glabrata*			*C. krusei*			*C. parapsilosis*		
	MIC_F_/MIC_C_	Gain	FICI	MIC_F_/MIC_C_	Gain	FICI	MIC_F_/MIC_C_	Gain	FICI	MIC_F_/MIC_C_	Gain	FICI
Tl	1/0.002	512	0.25 ^a^	1/0.002	512	0.25 ^a^	1/0.002	512	0.25 ^a^	1/0.002	512	0.25 ^a^
Ts	1/0.004	256	0.25 ^a^	1/0.004	256	0.25 ^a^	1/0.004	256	0.25 ^a^	1/0.016	64	0.27 ^a^
Tp	1/0.004	256	0.25 ^a^	1/0.004	256	0.25 ^a^	1/0.004	256	0.25 ^a^	1/0.016	64	0.27 ^a^
Oc	1/0.008	128	0,26 ^a^	1/0.008	128	0.26 ^a^	1/0.016	64	0.27 ^a^	1/0.062	16	0.31 ^a^
Al	1/0.008	128	0.26 ^a^	1/0.008	128	0.26 ^a^	1/0.031	32	0.28 ^a^	1/0.062	16	0.31 ^a^
Aha	1/0.016	64	0.27 ^a^	1/0.016	64	0.27 ^a^	1/0.016	64	0.27 ^a^	1/0.031	32	0.28 ^a^

MIC_F_/MIC_C_: MIC of Fluconazole alone/MIC of Fluconazole in combination with essential oil in mg/mL. ^a^ Synergism.

**Table 5:** Fractional inhibitory concentrations indices (FICIs) and gain of amphoterecin B combined with the essential oils.

EOs	*C. albicans*			*C. glabrata*			*C. krusei*			*C. parapsilosis*		
	MIC_A_/MIC_C_	Gain	FICI	MIC_A_/MIC_C_	Gain	FICI	MIC_A_/MIC_C_	Gain	FICI	MIC_A_/MIC_C_	Gain	FICI
Tl	0.0001/0.00005	2	0.75 ^b^	0.0001/0.00005	2	0.75 ^b^	0.0001/0.00003	4	0.50 ^a^	0.0004/0.0002	2	0.75 ^b^
Ts	0.0001/0.0001	1	1.25 ^c^	0.0001/0.0001	1	1.25 ^c^	0.0001/0.0001	1	1.25 ^c^	0.0004/0.0004	1	1.25 ^c^
Tp	0.0001/0.0001	1	1.25 ^c^	0.0001/0.0001	1	1.25 ^c^	0.0001/0.0001	1	1.25 ^c^	0.0004/0.0004	1	1.25 ^c^
Oc	0.0001/0.00005	2	0.75 ^b^	0.0001/0.00005	2	0.75 ^b^	0.0001/0.00003	4	0.50 ^a^	0.0004/0.0002	2	0.75 ^b^
Al	0.0001/0.0001	1	1.25 ^c^	0.0001/0.0001	1	1.25 ^c^	0.0001/0.0001	1	1.25 ^c^	0.0004/0.0004	1	1.25 ^c^
Aha	0.0001/0.00005	2	0.75 ^b^	0.0001/0.00005	2	0.75 ^b^	0.0001/0.00003	4	0.50 ^a^	0.0004/0.0002	2	0.75 ^b^

MIC_A_/MIC_C_: MIC of Amphoterecin B alone/MIC of Amphoterecin B in combination with essential oil in mg/mL. ^a^ Synergism; ^b^ Additive effect; ^c^ Indifference.

## Data Availability

Data is contained within the article.

## References

[1] Shapiro R.S., Robbins N., Cowen L.E. (2011). Regulatory circuitry governing fungal development, drug resistance, and disease. Microbiol. Mol. Biol. Rev..

[2] Anderson J. (2005). Evolution of antifungal-drug resistance: Mechanisms and pathogen fitness. Nat. Rev. Microbiol..

[3] Shao P.L., Huang L.M., Hsueh P.R. (2007). Recent advances and challenges in the treatment of invasive fungal infections. Int. J. Antimicrob. Agents.

[4] De Repentigny L., Lewandowski D., Jolicoeur P. (2004). Immunopathogenesis of oropharyngeal candidiasis in human immunodeficiency virus infection. Clin. Microbiol. Rev..

[5] Wisplinghoff H., Bischoff T., Tallent S., Seifert H., Wenzel R.P., Edmond M.B. (2004). Nosocomial bloodstream infections in US hospitals: Analysis of 24,179 cases from a prospective nation wide surveillance study. Clin. Infect. Dis..

[6] Filler S.G., Sheppard D.C. (2006). Fungal invasion of normally non-phagocytic host cells. PLoS Pathog..

[7] Nguyen M.H., Peacock J.E., Morris A.J., Tanner D.C., Nguyen M.L., Snydman D.R., Wagener M.M., Rinaldi M.G., Yu V.L. (1996). The changing face of candidemia: Emergence of non-*Candida albicans* species and antifungal resistance. Am. J. Med..

[8] Bojic-Milicevic G., Mikov M., Golocorbin-Kohn S. (2008). The importance of genus *Candida* in human samples. Zb. Matice Srp. Za Prir. Nauk..

[9] Prasad R., Shah A.H., Rawal M.K. (2016). Antifungals: Mechanism of action and drug resistance. Adv. Exp. Med. Biol..

[10] Lupetti A., Danesi R., Campa M., Del Tacca M., Kelly S. (2002). Molecular basis of resistance to azole antifungals. Trends Mol. Med..

[11] Spampinato C., Leonardi D. (2013). Candida infections, causes, targets, and resistance mechanisms: Traditional and alternative antifungal agents. BioMed Res. Int..

[12] Rex J.H., Rinaldi M.G., Pfaller M.A. (1995). Resistance of *Candida* species to fluconazole. Antimicrob. Agents Chemother..

[13] Herbrecht R., Natarajan-Amé S., Nivoix Y., Letscher-Bru V. (2003). The lipid formulations of amphotericin B. Expert Opin. Pharmacother..

[14] Ahmad A., Molepo J., Patel M. (2016). Challenges in the development of antifungal agents against *Candida*: Scope of phytochemical research. Curr. Pharm. Des..

[15] Ahmad A., Khan A., Manzoor N. (2013). Reversal of efflux mediated antifungal resistance underlies synergistic activity of two monoterpenes with fluconazole. Eur. J. Pharm. Sci..

[16] Soulaimani B., Nafis A., Kasrati A., Rochdi A., Mezrioui N.E., Abbad A., Hassani L. (2019). Chemical composition, antimicrobial activity and synergistic potential of essential oil from endemic *Lavandula maroccana* (Mill.). S. Afr. J. Bot..

[17] Azzimonti B., Cochis A., El Beyrouthy M., Iriti M., Uberti F., Sorrentino R., Landini M.M., Rimondini L., Varoni E.M. (2015). Essential oil from berries of Lebanese Juniperus excelsa M. Bieb displays similar antibacterial activity to chlorhexidine but higher cytocompatibility with human oral primary cells. Molecules.

[18] Ahmad A., Khan A., Khan L.A., Manzoor N. (2010). In vitro synergy of eugenol and methyleugenol with fluconazole against clinical *Candida* isolates. J. Med. Microbiol..

[19] Khan M.S.A., Malik A., Ahmad I. (2012). Anti-candidal activity of essential oils alone and in combination with amphotericin B or fluconazole against multi-drug resistant isolates of *Candida albicans*. Med. Mycol..

[20] Ahmad A., Khan A., Akhtar F., Yousuf S., Xess I., Khan L.A., Manzoor N. (2011). Fungicidal activity of thymol and carvacrol by disrupting ergosterol biosynthesis and membrane integrity against *Candida*. Eur. J. Clin. Microbiol. Infect. Dis..

[21] Khan A., Ahmad A., Akhtar F., Yousuf S., Xess I., Khan L.A., Manzoor N. (2010). *Ocimum sanctum* essential oil and its active principles exert their antifungal activity by disrupting ergosterol biosynthesis and membrane integrity. Res. Microbiol..

[22] Bellakhdar J. (1997). La Pharmacopée Marocaine Traditionnelle, Médicine Arabe Ancienne et Savoirs Populaire.

[23] El Abdouni Khayari M., Jamali C.A., Kasrati A., Hassani L., Leach D., Markouk M., Abbad A. (2016). Antibacterial activity of essential oils of some moroccan aromatic herbs against selected food-related bacteria. J. Essent. Oil-Bear. Plants.

[24] Alaoui Jamali C., Kasrati A., Fadli M., Hassani L., Leach D., Abbad A. (2017). Synergistic effects of three Moroccan thyme essential oils with antibiotic cefixime. Phytothérapie.

[25] Ouedrhiri W., Balouiri M., Bouhdid S., Moja S., Chahdi F.O., Taleb M., Greche H. (2016). Mixture design of *Origanum compactum*, *Origanum majorana* and *Thymus serpyllum* essential oils: Optimization of their antibacterial effect. Ind. Crop. Prod..

[26] Fadli M., Pagès J., Mezrioui N., Abbad A. (2016). *Artemisia herba-alba* Asso and *Cymbopogon citratus* (DC.) Stapf essential oils and their capability to restore antibiotics efficacy. Ind. Crop. Prod..

[27] Benchikha N., Rebiai A., Brahima O., Neghmouch N.S., Ben Amor M.L. (2019). Chemical composition, antimicrobial, antioxidant and anticancer activities of essential oil from *Ammodaucus leucotrichus* Cosson & Durieu (Apiaceae) growing in south Algeria. Bull. Chem. Soc. Ethiop..

[28] Soulaimani B., El Hidar N., Ben El Fakir S., Mezrioui N., Hassani L., Abbad A. (2021). Combined antibacterial activity of essential oils extracted from *Lavandula maroccana* (Murb.), *Thymus pallidus* Batt. and *Rosmarinus officinalis* L. against antibiotic-resistant Gram-negative bacteria. Eur. J. Integr. Med..

[29] Boubaker H., Karim H., El Hamdaoui A., Msanda F., Leach D., Bombarda I., Vanloot P., Abbad A., Boudyach E.H., Ait Ben Aoumar A. (2016). Chemical characterization and antifungal activities of four *Thymus* species essential oils against postharvest fungal pathogens of citrus. Ind. Crop. Prod..

[30] El Asbahani A., Jilale A., Voisin S.N., Aït Addi E.H., Casabianca H., El Mousadik A., Hartmann D.J., Renaud F.N.R. (2015). Chemical composition and antimicrobial activity of nine essential oils obtained by steam distillation of plants from the Souss-Massa Region (Morocco). J. Essent. Oil Res..

[31] Alaoui Jamali C., El Bouzidi L., Bekkouche K., Hassani L., Markouk M., Wohlmuthc H., Leach D., Abbad A. (2012). Chemical composition and antioxidant and anticandidal activities of essential oils from different wild moroccan *Thymus* species. Chem. Biodivers..

[32] Fadli M., Chevalier J., Saad A., Mezrioui N.E., Hassani L., Pages J.M. (2011). Essential oils from Moroccan plants as potential chemosensitisers restoring antibiotic activity in resistant Gram-negative bacteria. Int. J. Antimicrob. Agents.

[33] Ghalbane I., Belaqziz R., Ait Said L., Oufdou K., Romane A., El Messoussi S. (2011). Chemical composition, antibacterial and antioxidant activities of the essential oils from *Thymus satureioides* and *Thymus pallidus*. Nat. Prod. Commun..

[34] Laghmouchi Y., Belmehdi O., Senhaji N.S., Abrini J. (2018). Chemical composition and antibacterial activity of Origanum *compactum* Benth. essential oils from different areas at northern Morocco. S. Afr. J. Bot..

[35] Bouyahya A., Dakka N., Talbaoui A., Et-Touys A., El-Boury H., Abrini J., Bakri Y. (2017). Correlation between phenological changes, chemical composition and biological activities of the essential oil from Moroccan endemic Oregano (*Origanum compactum* Benth). Ind. Crop. Prod..

[36] El Bouzidi L., Jamali C.A., Bekkouche K., Hassani L., Wohlmuth H., Leach D., Abbad A. (2013). Chemical composition, antioxidant and antimicrobial activities of essential oils obtained from wild and cultivated Moroccan *Thymus* species. Ind. Crop. Prod..

[37] Chraibi M., Farah A., Lebrazi S., El Amine O., Iraqui Houssaini M., Fikri-Benbrahim K. (2016). Antimycobacterial natural products from Moroccan medicinal plants: Chemical composition, bacteriostatic and bactericidal profile of *Thymus satureioides* and *Mentha pulegium* essential oils. Asian Pac. J. Trop. Biomed..

[38] Sadaoui N., Bec N., Barragan-Montero V., Kadri N., Cuisinier F., Larroque C., Arab K., Khettal B. (2018). The essential oil of Algerian *Ammodaucus leucotrichus* Coss. & Dur. and its effect on the cholinesterase and monoamine oxidase activities. Fitoterapia.

[39] Khaldi A., Meddah B., Moussaoui A., Sonnet P. (2017). Anti-mycotoxin effect and antifungal properties of essential oil from *Ammodaucus leucotrichus* Coss. & Dur. on *Aspergillus flavus* and *Aspergillus ochraceus*. J. Essent. Oil-Bear. Plants.

[40] Manssouri M., Znini M., El Harrak A., Majidi L. (2016). Antifungal activity of essential oil from the fruits of *Ammodaucus leucotrichus* Coss. & Dur., in liquid and vapour phase against postharvest phytopathogenic fungi in apples. J. Appl. Pharm. Sci..

[41] Abu-darwish M.S., Cabral C., Gonçalves M.J., Cavaleiro C., Cruz M.T. (2015). *Artemisia herba-alba* essential oil from Buseirah (South Jordan): Chemical characterization and assessment of safe antifungal and anti-in fl ammatory doses. J. Ethnopharmacol..

[42] Bertella A., Benlahcen K., Abouamama S., Pinto D.C.G.A., Maamar K., Kihal M., Silva A.M.S. (2018). *Artemisia herba-alba* Asso. essential oil antibacterial activity and acute toxicity. Ind. Crop. Prod..

[43] Amor G., Caputo L., La Storia A., De Feo V., Mauriello G., Fechtali T. (2019). *Artemisia herba-alba* and *Origanum majorana* Essential Oils from Morocco. Molecules.

[44] Mohammedi H., Idjeri-Mecherara S., Menaceur F., Azine K., Hassani A. (2018). Chemical compositions of extracted volatile oils of *Ammodaucus leucotrichus* L. fruit from different geographical regions of Algeria with evaluation of its toxicity, anti-inflammatory and antimicrobial activities. J. Essent. Oil-Bear. Plants.

[45] Louail Z., Kameli A., Benabdelkader T., Bouti K., Hamza K., Krimat S. (2016). Antimicrobial and antioxidant activity of essential oil of *Ammodaucus leucotrichus* Coss. & Dur. seeds. J. Mater. Environ. Sci..

[46] El-Haci I., Bekhechi C., Atik-Bekkara F., Mazari W., Gherib M., Bighelli A., Csanova J., Tomi F. (2014). Antimicrobial activity of *Ammodaucus leucotrichus* fruit oil from algerian sahara. Nat. Prod. Commun..

[47] Mcgeady P., Wansley D.L., Logan D.A. (2002). Filamentous structures in *Candida* significant inhibition of growth. J. Nat. Prod..

[48] Tian J., Wang Y., Zeng H., Li Z., Zhang P., Tessema A., Peng X. (2015). Efficacy and possible mechanisms of perillaldehyde in control of *Aspergillus niger* causing grape decay. Int. J. Food Microbiol..

[49] Tian J., Zeng X., Lü A., Zhu A., Peng X., Wang Y. (2015). Perillaldehyde, a potential preservative agent in foods: Assessment of antifungal activity against microbial spoilage of cherry tomatoes. LWT—Food Sci. Technol..

[50] Asdadi A., Alilou H., Akssira M., Mina L., Hassani I., Chebli B. (2014). Chemical composition and anticandidal effect of three *Thymus* species essential oils from southwest of Morocco against the emerging nosocomial fluconazole-resistant strains. Biol. Agric. Healthc..

[51] Burt S.A., Vlielander R., Haagsman H.P., Veldhuizen E.J.A. (2005). Increase in activity of essential oil components carvacrol and thymol against *Escherichia coli* O157:H7 by addition of food stabilizers. J. Food Prot..

[52] Lambert R.J., Skandamis P., Coote P., Nychas G.-J. (2001). A study of the minimum inhibitory concentration and mode of action of oregano essential oil, thymol and carvacrol. J. Appl. Microbiol..

[53] Saad N.Y., Muller D., Lobstein A. (2013). Major bioactivities and mechanism of action of essential oils and their components. Flavour Fragr. J..

[54] Rajput S.B., Mohan Karuppayil S. (2013). Small molecules inhibit growth, viability and ergosterol biosynthesis in *Candida albicans*. Springerplus.

[55] Palaniappan K., Holley R.A. (2010). International Journal of Food Microbiology Use of natural antimicrobials to increase antibiotic susceptibility of drug resistant bacteria. Int. J. Food Microbiol..

[56] Di Pasqua R., Betts G., Hoskins N., Edwards M., Ercolini D., Mauriello G. (2007). Membrane toxicity of antimicrobial compounds from essential oils. J. Agric. Food Chem..

[57] Ultee A., Bennik M.H.J., Moezelaar R. (2002). The phenolic hydroxyl group of carvacrol is essential for action against the food-borne pathogen *Bacillus cereus*. Appl. Environ. Microbiol..

[58] Adams R.P. (2007). Identification of Essential Oil Coponents by Gaz Chromatography.

[59] Saad A., Fadli M., Bouaziz M., Benharref A., Mezrioui N.E., Hassani L. (2010). Anticandidal activity of the essential oils of *Thymus maroccanus* and *Thymus broussonetii* and their synergism with amphotericin B and fluconazol. Phytomedicine.

[60] Clinical and Laboratory Standards Institute (CLSI) (2008). Reference Method for Broth Dilution Antifungal Susceptibility Testing of Yeasts. Approved Standard M27-A3.

[61] European Commitee for Antimicrobial Susceptibility Testing (EUCAST) (2000). Terminology relating to methods for the determination of susceptibility of bacteria to antimicrobial agents. Clin. Microbiol. Infect..

